# C-Terminus of Progranulin Interacts with the Beta-Propeller Region of Sortilin to Regulate Progranulin Trafficking

**DOI:** 10.1371/journal.pone.0021023

**Published:** 2011-06-15

**Authors:** Yanqiu Zheng, Owen A. Brady, Peter S. Meng, Yuxin Mao, Fenghua Hu

**Affiliations:** Department of Molecular Biology and Genetics, Weill Institute for Cell and Molecular Biology, Cornell University, Ithaca, New York, United States of America; Tokyo Medical and Dental University, Japan

## Abstract

Progranulin haplo-insufficiency is a main cause of frontotemporal lobar degeneration (FTLD) with TDP-43 aggregates. Previous studies have shown that sortilin regulates progranulin trafficking and is a main determinant of progranulin level in the brain. In this study, we mapped the binding site between progranulin and sortilin. Progranulin binds to the beta-propeller region of sortilin through its C-terminal tail. The C-terminal progranulin fragment is fully sufficient for sortilin binding and progranulin C-terminal peptide displaces progranulin binding to sortilin. Deletion of the last 3 residues of progranulin (QLL) abolishes its binding to sortilin and also sortilin dependent regulation of progranulin trafficking. Since progranulin haplo-insufficiency results in FTLD, these results may provide important insights into future studies of progranulin trafficking and signaling and progranulin based therapy for FTLD.

## Introduction

Frontotemporal lobar degeneration (FTLD) is one of the most prevalent forms of early onset dementia (45–65 years of age), second only to Alzheimer's disease [1], [2]. Clinical features of FTLD include memory deficits, behavioral abnormalities, personality changes and language impairments [3]. Mutations in the *Progranulin (PGRN)* gene were recently shown to be a common cause of FTLD and the major cause of FTLD with tau-negative ubiquitin positive inclusions [4], [5], [6]. So far 68 distinct PGRN mutations have been found in 229 families (http://www.molgen.ua.ac.be/FTDMutations), responsible for 5–10% of all FTLD cases. Most mutations result in a decrease in the amount of progranulin expressed or secreted, rather than a gain of toxicity [4], [5]. Thus progranulin haplo-insufficiency is strongly associated with FTLD [4], [5].

Progranulin is an evolutionarily conserved, secreted glycoprotein that can be proteolytically processed into several granulin peptides (granulins A, B, C, D, E, F, G) [7]. It regulates cell proliferation, cell mobility, inflammation and wound healing [8], [9]. The normal functions of progranulin in the central nervous system (CNS) remain to be defined, although studies have suggested a role of progranulin in promoting neuronal survival and regulating inflammation in the CNS [10], [11], [12], [13], [14], [15]. Moreover, mechanisms that regulate progranulin levels and the receptor(s) and signaling pathways involved in progranulin action remain to be defined.

Sortilin was recently identified as a progranulin binding partner in an expression cloning screen [16]. Sortilin is a type I single pass transmembrane protein in the VPS10 family which regulates intracellular protein trafficking and acts as a cell surface receptor that mediates pro-NGF and pro-BDNF mediated cell death when coupled with p75/NTR [17]. Sortilin mediates progranulin endocytosis and regulates the level of progranulin in the brain [16]. The level of secreted progranulin is dramatically increased in sortilin knockout mice [16]. Furthermore, ablation of sortilin is able to correct the decreased progranulin level in mice heterozygous for *PGRN* deletion [16]. A genome wide association study has also found two SNPs close to sortilin that affect sortilin expression associated with PGRN level in the plasma [18]. Thus progranulin-sortilin interaction is a major determinant of progranulin level *in vivo*.

Here we report the mapping of the binding sites between progranulin and sortilin. We show that progranulin binds to the beta propeller region of sortilin through its C-terminal tail. The crystal structure of the VPS10 domain of sortilin was recently determined in a complex with another sortilin ligand, neurotensin [19]. Neurotensin, a brain-gut tridecapeptide, interacts with the sortilin beta-propeller domain via its extreme carboxyl terminus. Our data suggests that progranulin and neurotensin interact with sortilin in a similar fashion.

## Results

### Progranulin binds to the beta-propeller region of sortilin

**Figure 1:**
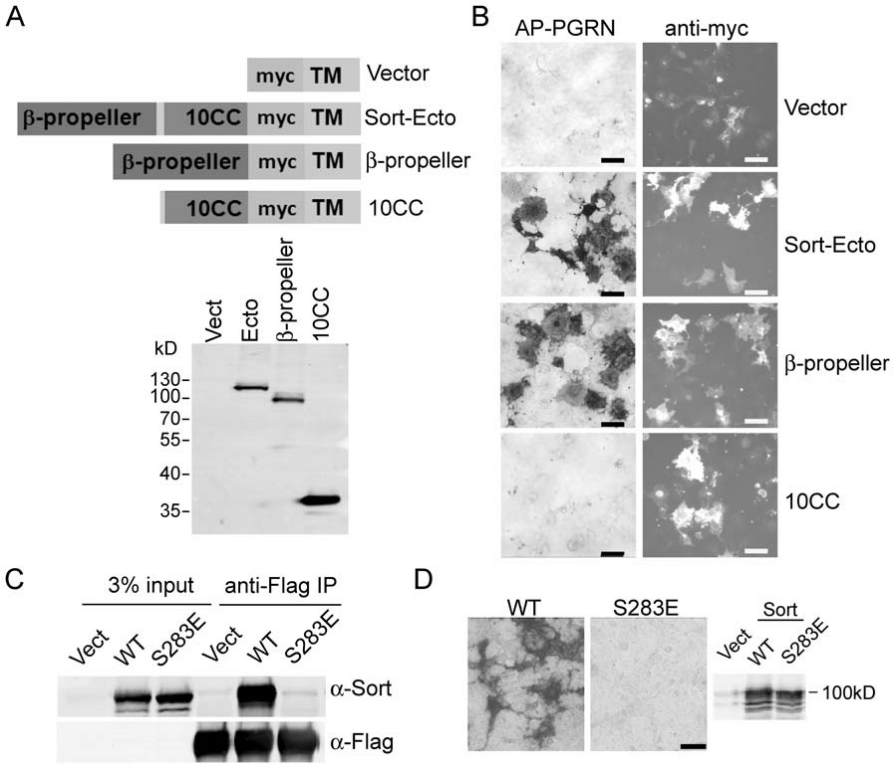
Progranulin interacts with the beta-propeller region of sortilin. (**A**) A schematic drawing of sortilin fragments cloned in the pDisplay vector (full length sortilin ecto domain (residues 81–757), sortilin 10CC region (residues 612–757) or sortilin beta propeller domain (residues 81–611)) and western blot to confirm their expression levels in COS-7 cells using anti-myc antibodies. (**B**) Binding of 100 nM alkaline phosphatase tagged progranulin (AP-PGRN) to COS-7 cells expressing various sortilin construct as indicated. Expression of these constructs was verified by anti-myc staining. (**C**) S283E mutation of sortilin abolished progranulin binding. Cell lysates from HEK293T cells transfected with vector control, wild type sortilin or the S283E mutant of sortilin were incubated with anti-Flag beads bound to Flag-PGRN. The presence of sortilin in the 3% input and anti-Flag IP was detected using anti-sortilin antidodies. (**D**) COS-7 cells expressing wild type sortilin or sortilin S283E mutant were allowed to bind to 100 nM AP-PGRN. The expression of S283E mutant was confirmed with western blot using anti-sortilin antibodies. Scale bar = 100 µm.

Sortilin is a multi-functional receptor that interacts with an increasing number of ligands [17]. The VPS10 domain of sortilin consists of two structural modules, a ten-bladed beta-propeller structure at the N-terminus (residues 81–611) and a cysteine rich 10CC module (10CCa and 10CCb, residues 612–757) at the C-terminus [19]. To determine which structural domain of sortilin is involved in progranulin binding, we expressed the beta-propeller region or the 10CC region of sortilin fused to the transmembrane domain of PDGFR. While the full length VPS10 domain and beta-propeller region of sortilin could support progranulin binding when expressed on the cell surface, the 10CC domain failed to bind to progranulin. The beta-propeller and 10CC regions showed similar expression levels in the cell by both immunofluorescence staining and western blot (Fig. 1A and 1B). This suggests that progranulin interacts with the beta-propeller domain of sortilin but not the 10CC region.

Two modes of interaction between sortilin and its ligands have been demonstrated. The C-terminal residues of neuropeptide neurotensin (PYIL) bind inside the tunnel of the beta-propeller, interacting with blades 5–6 in the beta-propeller region of VPS10 [19]. Serine 283 of sortilin makes hydrogen bonds with the C-terminal carboxylate of neurotensin through its side chains [19]. S283E mutation abolishes neurotensin binding to sortilin [19]. On the other hand, the pro-domains of nerve growth factor (NGF) and brain derived neurotrophic factor (BDNF) bind to sortilin through a linear surface exposed sequence [20] and the S283E mutation of sortilin has no effect on pro-NGF or pro-BDNF binding [19]. We found that the S283E mutation in the beta-propeller region of sortilin abolishes progranulin binding (Fig. 1C and 1D), suggesting that progranulin binds to sortilin in a manner similar to neurotensin.

### The C-terminus of progranulin mediates its binding to sortilin

**Figure 2:**
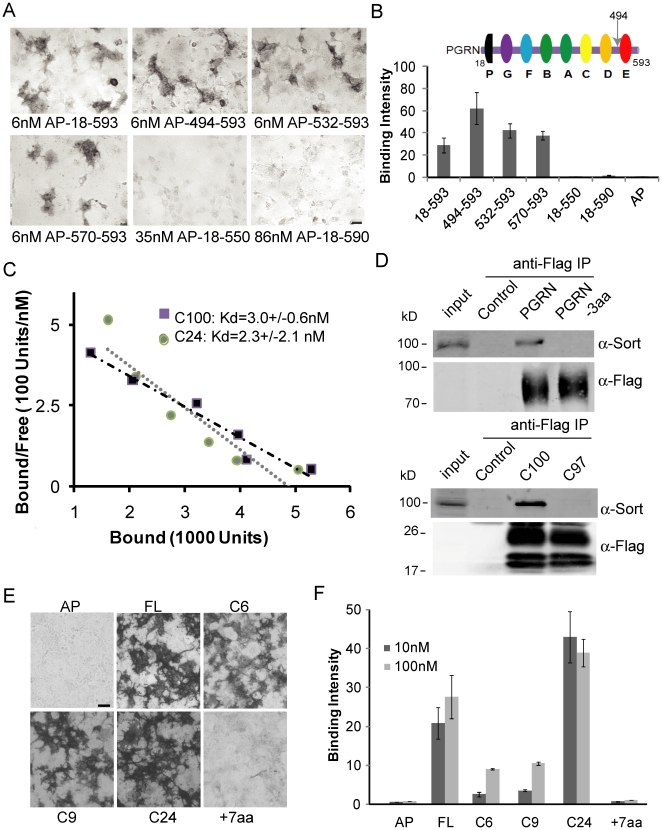
Progranulin C-terminus mediates its binding to sortilin. (**A**) COS-7 cells expressing wild type sortilin were allowed to bind AP tagged progranulin fragments as indicated. (**B**) Quantification of the binding assay as described in (A). The intensity of AP staining was measured using the Metaexpress software (Y axis: average integrated optical density per cell (×100)). (**C**) C24 fragment of progranulin binds to sortilin with a similar affinity as the C100 fragment and full length progrnaulin. Sortilin expressing COS-7 cells were allowed to bind to AP tagged C100 and C24 at increasing concentrations. Scatchard plot was used to determine the Kd of the binding. (**D**) Flag tagged PGRN and C100 but not PGRNΔ3aa or C97 pull down sortilin in the cell lysates of NSC-34, a motor neuron cell line. (**E**) Alkaline phosphatase fused full length PGRN, C-terminal C100, C9 or C6 proteins binds to sortilin expressed in COS-7 cells at 100 nM, but not full length PGRN protein with additional 7 residues (GPMHETR) at its C-terminus. (**F**) Quantification of binding intensity for the experiment in (E) using the MetaXpress software. Scale bar = 100 µm.

In order to determine regions in progranulin responsible for sortilin binding, we made a series of deletion constructs. Granulin E and its flanking region (C100, aa 494–593) have been shown to be fully sufficient for binding to sortilin while the rest of the mature progranulin protein (aa 18–494) does not bind to sortilin [16]. Since the last three residues of progranulin (QLL) are similar to those of neurotensin (YIL), we propose that progranulin might interact with sortilin through its C-terminal residues like neurotensin. Indeed, we found that deletion of the last 3aa of progranulin (residues QLL) (PGRNΔ3aa) abolishes progranulin binding to sortilin in the COS-7 binding assay (Fig. 2A and 2B) and in the co-immunoprecipitation experiment (Fig. 2D), supporting a critical role of these residues in mediating progranulin-sortilin interaction. To determine whether the C-terminal tail of progranulin is sufficient for sortilin binding, we made N-terminal alkaline phosphatase (AP) fusion proteins with the last 24, 9 or 6 residues of progranulin. We found that the last 24aa of progranulin (C24, aa 570–593) is fully sufficient for binding to sortilin (Fig. 2A–C). Alkaline phosphatase tagged C24 binds to sortilin with a Kd in the nM range, similar to C100 and full length progranulin [16] (Fig. 2C). The fusion of the C-terminal 9 residues (C9) or 6 residues (C6) of progranulin to AP also enables the fusion protein to interact with sortilin, suggesting that the last 6 residues (C6, ALRQLL) are able to mediate progranulin-sortilin interaction, although not to the same extent as the 24 residues (Fig. 2E and 2F). Steric hindrance due to the AP fusion might reduce the flexibility and binding affinity of the C9 and C6 peptide to sortilin. The C-terminal carboxylate of leucine residue was shown to be critical for neurotensin-sortilin binding [19]. We found that addition of residues to the progranulin C-terminus (+7aa) abolishes the binding of progranulin to sortilin, further confirming a critical role of progranulin's C-terminal leucine residue in mediating its binding to sortilin (Fig. 2E, 2F). Western blot analysis showed proper molecular weight for all the AP-fusion proteins used in our study (Fig. S1).

**Figure 3:**
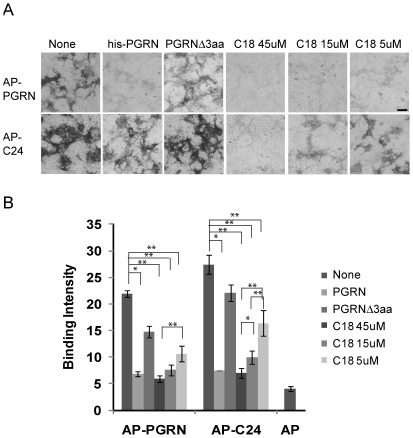
Progranulin C-terminal peptide displaces progranulin binding to sortilin. (**A**) Alkaline phosphatase fused full length PGRN and C24 binding to sortilin can be displaced by purified recombinant his-PGRN, C18 peptide (EAPRWDAPLRDPALRQLL) but not by his-PGRNΔ3aa. Sortilin expressing COS-7 cells were incubated with 150 nM purified his-PGRN, his-PGRNΔ3aa or C18 peptide of indicated concentrations for 1 hour before adding 10 nM of AP-PGRN or AP-C24. (**B**) Quantification of binding intensity for experiments in (A) using the ImageXpress system. Scale bar = 100 µm. *, p<0.05;**, p<0.01, n = 3–6, paired Student's T-test.

Furthermore, binding of alkaline phosphatase tagged C24 fragment (AP-C24) and full length progranulin (AP-PGRN) to sortilin can be efficiently displaced by recombinant histidine tagged full length progranulin (his-PGRN) and the C18 peptide C-terminal to the granulin E motif (Fig. 3A and 3B). Deletion of the last 3 residues from the recombinant his-PGRN protein (PGRNΔ3aa) dramatically reduced its ability to compete with AP-PGRN for binding to sortilin (Fig. 3). The C-terminal 6 residues of neurotensin also displace binding of progranulin to sortilin [16]. These data further support that the C-terminal tail of progranulin is responsible for progranulin-sortilin interaction and progranulin binds to sortilin in a manner similar to neurotensin.

### Conservation of sortilin binding motifs in progranulin

**Figure 4:**
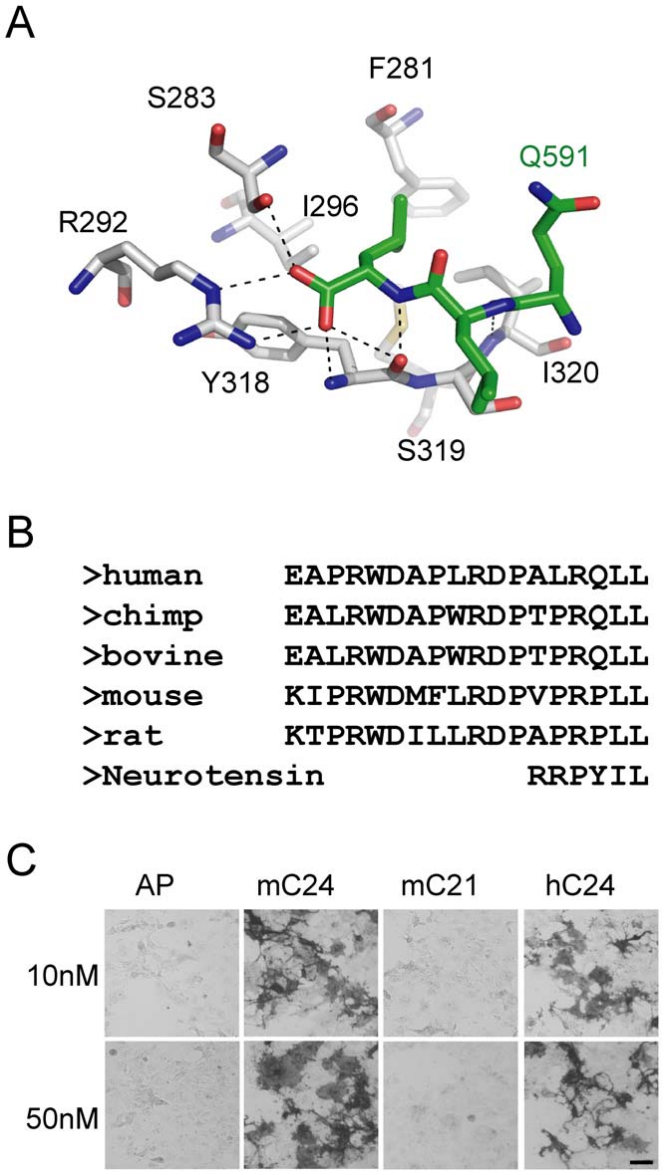
The C-terminal carboxylate of progranulin mediates its binding to sortilin. (**A**) Illustration of binding of progranulin C-terminal tail to sortilin. Drawing was made with Pymol software. Residue backbones of sortilin are labeled in gray and progranulin labeled in green. (**B**) Alignment of the C-terminal residues of progranulin from different species of mammals (from top to bottom: gi|4504151|[Homo sapiens], gi|114666849|[Pan troglodytes], gi|296476263|[Bos taurus], gi|224967126|[Mus musculus], gi|204224|[Rattus norvegicus] ) and neurotensin. (**C**) Mouse progranulin interacts with mouse sortilin through its C-terminal tail. COS-7 cells transfected with mouse sortilin were incubated with 10 or 50 nM of AP tagged C24 and C21 of mouse progranulin (mC24 and mC21) or C24 of human progranulin (hC24). Scale bar = 100 µm.

We modeled progranulin binding to sortilin according to the co-crystal structure of sortilin and neurotensin. Like neurotensin, the C-terminal carboxylate of progranulin makes most contacts with the beta-propeller structure of sortilin (Fig. 4A). The C-terminal leucine fits into a hydrophobic pocket of sortilin surrounded by residues F281, S283, R292, Y318 and S319, forms a salt bridge with the guanidinium group of R292 and makes hydrogen bonds with the side chain of S283 and the main chain amide of Y318. Alignment of progranulin sequences from mammals revealed the conservation of the C-terminal leucine residue (Fig. 4B). However, it seems there is no absolute requirement for other residues for sortilin binding besides the C-terminal leucine. Deletion of the last 3 residues (PLL) in the C-terminal 24 residues of mouse progranulin abolishes its binding to sortilin, supporting that PLL in mouse progranulin mediates its binding to sortilin (Fig. 4C). Thus YIL in neurotensin, QLL in human progranulin as well as PLL in mouse progranulin all mediate binding to sortilin, suggesting a great flexibility in the residue requirement for sortilin interaction other than the C-terminal leucine as long as the C-terminal tail of the protein can fit into the hydrophobic pocket of sortilin.

### Sortilin regulated progranulin trafficking is abolished by C-terminal deletion of progranulin

**Figure 5:**
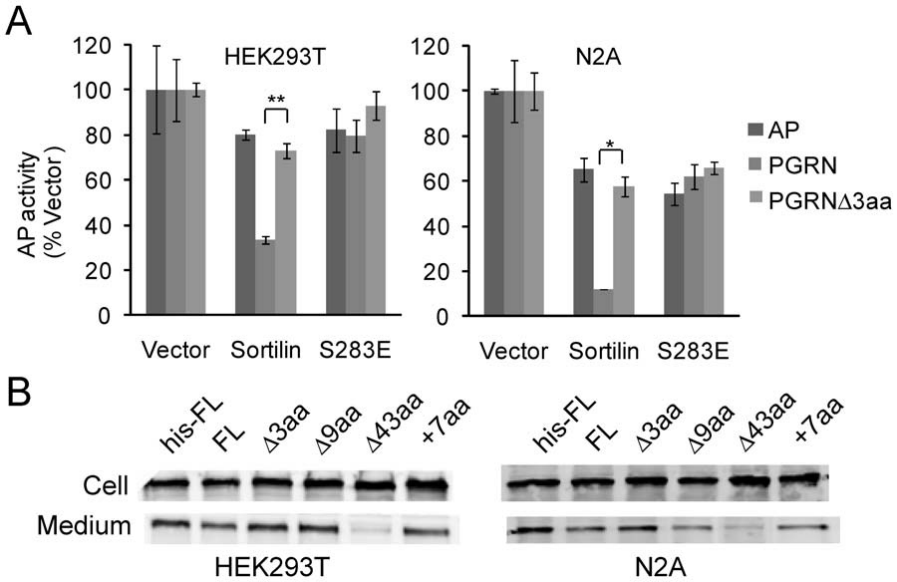
Deletion of progranulin C-terminal tail abolishes sortilin dependent control of progranulin trafficking but does not affect progranulin secretion. (**A**) Deletion of the PGRN C-terminal 3 residues (PGRNΔ3aa) or S283E mutation in sortilin abolishes sortilin dependent control of PGRN trafficking. AP tagged full length progranulin (FL), PGRNΔ3aa (-3aa) or AP alone were co-transfected with pcDNA vector control, wild type or S283E mutant of sortilin into HEK 293T cells or N2A cells. The AP activities in the medium were measured 4 days after transfection. The AP activities in sortilin expressing cells were expressed as percentage of vector control. *, p<0.05;**, p<0.01, n = 3–5, paired Student's T-test. (**B**) Deletion of progranulin C-terminal residues does not affect progranulin secretion. Progranulin expression constructs as AP fusion proteins were transfected in HEK293T and N2Acells. Progranulin levels in the cell lysates and media were determined by western blots using anti-AP antibodies.

Sortilin reduces the extracellular level of progranulin, possibly through active endocytosis and sorting in the TGN [16]. When sortilin is overexpressed in HEK293T cells, the extracellular level of progranulin is dramatically reduced compared to vector control [16] (Fig. 5A). However, when PGRNΔ3aa is co-transfected with sortilin or when full length PGRN is co-transfected with S283E mutant of sortilin, this effect of sortilin on progranulin is totally abolished (Fig. 5A). The same effect was observed in the neuroblastoma cell line N2A (Fig. 5A). Thus progranulin is no longer subject to sortilin mediated trafficking when the interaction of PGRN and sortilin is abolished by deleting the last 3 residues of progranulin or mutating the serine 283 to glutamate in sortilin.

Although the progranulin C-terminal tail is critical for sortilin interaction, it is not required for its secretion in the constitutive secretion pathway. When transfected into HEK293T cells or N2A cells, deletions or additions in the C-terminal tail that abolish progranulin binding to sortilin do not affect progranulin secretion into the medium (Fig. 5B), while deletion of part of granulin E segment (-43aa) results in retention of progranulin inside of the cell, suggesting that the progranulin C-terminal tail is not required for constitutive progranulin secretion.

## Discussion

In this study, we show that the binding between progranulin and sortilin is mediated by the C-terminal tail of progranulin and the beta-propeller region of sortilin. Progranulin binds to sortilin in a manner similar to neurotensin: (1) S283E mutation of sortilin abolishes progranulin binding, as well as neurotensin binding, but does not affect pro-neurotrophin binding; (2) Progranulin C-terminal tail is sufficient to mediate sortilin interaction and the last 3 residues of progranulin (QLL) are required for binding to sortilin, which are similar to the amino acid composition of neurotensin C-terminal residues (YIL).

Since progranulin haplo-insufficiency is a leading cause of FTLD and sortilin is a main determinant of progranulin level, reagents that modulate progranulin-sortilin interaction and thus help restore progranulin levels in the brain might be of high therapeutic interest. Our study demonstrated a critical role of the progranulin C-terminal tail in mediating its interaction with sortilin and thus an important function of this fragment in controlling progranulin trafficking. This result will impact future experiments with progranulin gene therapy or protein administration to treat FTLD. By deleting the last 3 residues of progranulin, higher levels of progranulin in the brain could be possibly achieved by bypassing sortilin mediated regulation of progranulin trafficking and lysosomal degradation. However, it is still not clear whether the progranulin-sortilin interaction also plays a role in progranulin signaling. Future studies are required to determine whether sortilin mediates the reported effects of progranulin on neuronal survival and inflammation. We have also found that the C-terminal carboxylate of progranulin in crucial in mediating the interaction between progranulin and sortilin. Addition of 7 residues at the C-terminus abolished progranulin binding to sortilin (Fig. 2E). Thus C-terminal tagging of progranulin should be avoided in order to preserve the interaction between progranulin and sortilin.

Since the minimum requirement for sortilin binding is fairly degenerate, our study also raises the question about the specificity of the progranulin-sortilin interaction. Progranulin does not interact with other mammalian sortilin homologs, such as sorLA and sorCS1 [16], suggesting that the specific organization of the sortilin beta-propeller region is required for this binding to occur. Sortilin is a multi-functional receptor with many identified ligands. Some of the ligands, such as cathepsin D also have C-terminal leucine residues (gi|179948, human cathepsin D, ARL and gi|817945, mouse cathepsin D, VVL). While it remains to be determined whether cathepsin D interacts with sortilin through its C-terminal leucine residue, there might be other unknown ligands for sortilin that bind through similar mechanisms.

The C-terminal peptide of progranulin is the only binding site for sortilin in progranulin. The granulin motif does not bind to sortilin and possesses unique biological activities in proliferation, inflammation and wound healing [7], [8], [9]. This indicates there exist other receptors that mediate the effects of progranulin or its cleaved products, granulin peptides. In this regard, a recent study demonstrated direction interactions between progranulin and tumor necrosis factor receptors [21].

Our study also suggests that progranulin and pro-neurotrophins binds to sortilin through different sites. The pro-domain of nerve growth factor (NGF) and brain derived neurotrophic factor (BDNF) binds to sortilin through a linear surface exposed sequence [20] rather than interacting inside of the beta-propeller tunnel like neurotensin and progranulin. This binding motif is consistent with the published data that progranulin and pro-NGF can bind to sortilin simultaneously in a Surface Plasmon Resonance experiment [16]. However, neurotensin blocks pro-neurotrophin induced neuronal cell death through sortilin although it binds to a different site. High levels of pro-NGF could also displace progranulin binding to sortilin [16]. These data suggest that although progranulin and pro-neurotrophins interact with sortilin through different mechanisms, steric hindrance and possible effects on the assembly of the ligand-receptor signaling complex is still likely to happen with regards to progranulin affecting pro-neurotrophin binding to sortilin and signaling.

## Materials and Methods

### Plasmids

To generate AP fusion proteins, human progranulin coding sequence for corresponding fragments were amplified and ligated to the pAP5-tag vector (GenHunter, Nashville, TN) digested with restriction enzymes XhoI and PmeI or XhoI and ApaI. Plasmids were then transfected into HEK293T cells and conditioned media were collected after 5 days.

To generate fusion protein of sortilin Ecto domain with the platelet derived growth factor receptor (PDGFR) transmembrane domain, sortilin Ecto domain (aa 81–757), 10CC region (aa 612–757) or beta-propeller region (aa 81–611) were cloned into the pDisplay vector (Invitrogen) using SfiI and XmaI sites. S283E was generated using site directed mutagenesis using the sortilin cDNA from Origene as the template.

### Cell culture procedures

NSC34 [22], N2A, HEK293T and COS-7 cells were grown in DMEM supplemented with 10% FBS, 1% Penicillin-Streptomycin at 37°C in a 5% CO_2_ atmosphere. All cell lines were obtained from ATCC. Cells were transiently transfected with polyethlyeneimine (PEI) as described [23].

### Binding assays

COS-7 binding assays were done as described [16]. Conditioned media with AP ligands were incubated with untransfected or sortilin transfected COS-7 cells 2 hrs at room temperature before fixation and heat inactivation of endogenous AP. Bound AP to COS-7 cells was measured using the ImageXpress imaging system (Molecular Devices).

### Antibodies

The following antibodies were used in the study: mouse anti-sortilin antibodies from BD biosciences, goat anti-sortilin antibodies from R&D systems, goat anti-alkaline phosphatase antibodies from Santa Cruz, rabbit anti-progranulin antibodies from Zymed, and mouse anti-myc (9E10) antibodies from Sigma-Aldrich.

### Immunoprecipitation

Cells were lysed in 50 mM Tris, pH 8.0, 150 mM NaCl, 1%Triton, 0.1% sodium deoxycholate and proteinase inhibitors (Roche). Mouse anti-Flag conjugated beads (Sigma) were allowed to bind to Flag-PGRN from the conditioned media of transfected HEK293T cells. These beads were incubated with cell lysates for 4 hours at 4°C and washed 4 times with the lysis buffer.

### Western-blot analysis

Protein samples in SDS sample buffer containing β-mercaptoethanol were boiled 10 min and centrifuged 1 min at 12,000 rpm. Samples were run on 12% polyacrylamide gels and transferred to Immobilon-FL PVDF membranes (Millipore). Membranes were blocked with 5% non-fat milk in PBS for 1 hour followed by incubation with primary antibodies for 2 hr at room temperature or overnight at 4°C. Membranes were washed for 5 min three times in Tris-Buffered Saline with 0.1% Tween-20 (TBS-T), incubated with secondary antibody for 2 hr at room temperature and washed three more times with TBS-T. Blots were imaged and quantified using an Odyssey Infrared Imaging System (LI-COR Biosciences).

## Supporting Information

Figure S1 **Western blot of AP fusion proteins used in the study.** Conditioned medium collected from transfected HEK293T cells containing indicated progranulin fragments as AP fusion proteins were subject to SDS-PAGE and western blot using anti-AP antibodies.
(TIF)
