# Supplementary figures and images for: C-Terminus of Progranulin Interacts with the Beta-Propeller Region of Sortilin to Regulate Progranulin Trafficking
Source: PLoS One. 2011 Jun 15;6(6):e21023. doi: 10.1371/journal.pone.0021023 (PMC3115958; doi:10.1371/journal.pone.0021023)

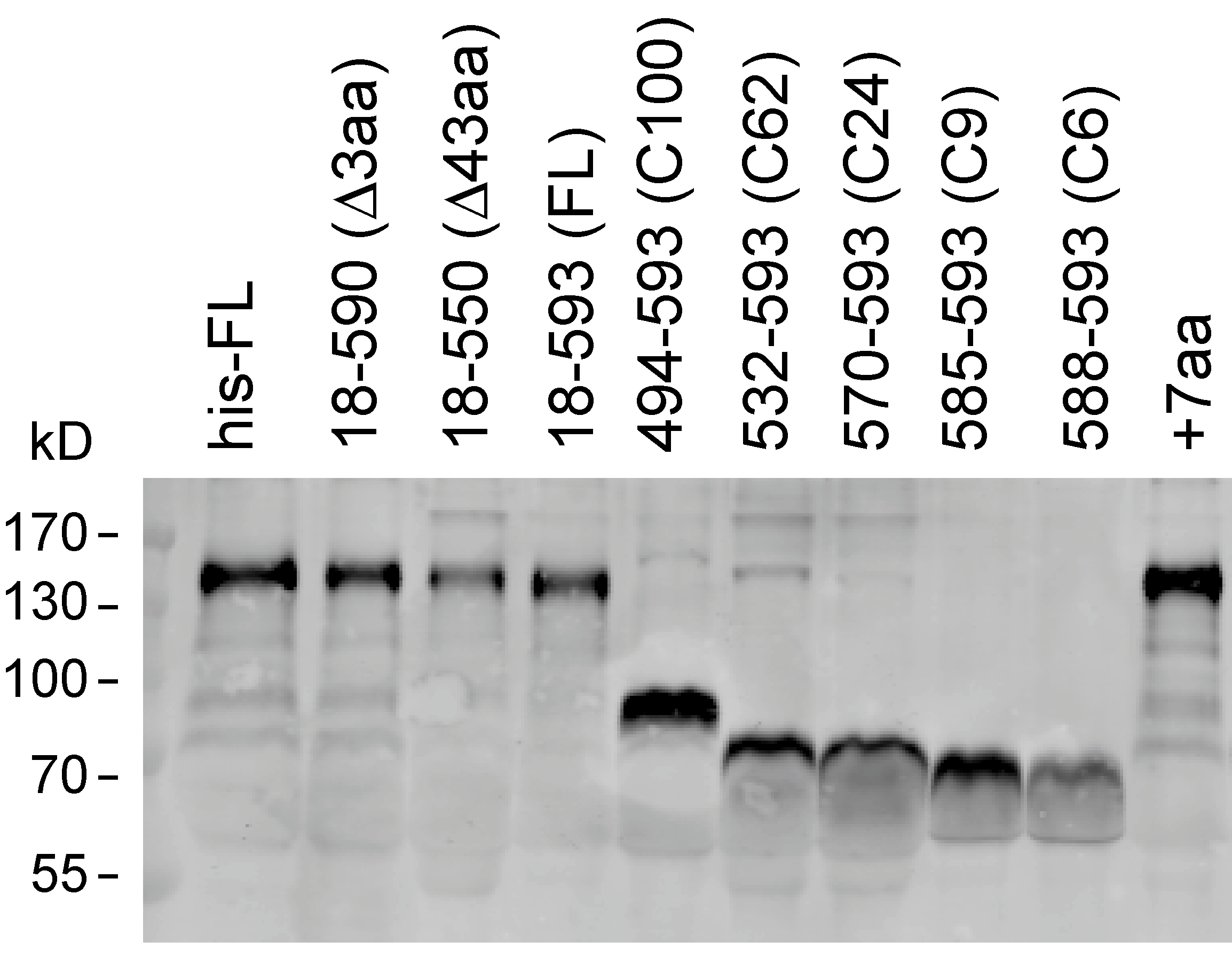

Supplement: Figure S1 — Western blot of AP fusion proteins used in the study. Conditioned medium collected from transfected HEK293T cells containing indicated progranulin fragments as AP fusion proteins were subject to SDS-PAGE and western blot using anti-AP antibodies. (TIF) [file pone.0021023.s001.tif]
